# Impact of Legumes as a Pre-Crop on Nitrogen Nutrition and Yield in Organic Greenhouse Tomato

**DOI:** 10.3390/plants10030468

**Published:** 2021-03-02

**Authors:** Anastasios Gatsios, Georgia Ntatsi, Luisella Celi, Daniel Said-Pullicino, Anastasia Tampakaki, Dimitrios Savvas

**Affiliations:** 1 Laboratory of Vegetable Crops, Department of Crop Science, Agricultural University of Athens, 11855 Athens, Greece; gatsios@aua.gr (A.G.); ntatsi@aua.gr (G.N.); 2 Department of Agricultural, Forest and Food Sciences, University of Torino, 10095 Grugliasco, TO, Italy; luisella.celi@unito.it (L.C.); daniel.saidpullicino@unito.it (D.S.-P.); 3 Laboratory of General and Agricultural Microbiology, Department of Crop Science, Agricultural University of Athens, 11855 Athens, Greece; tampakaki@aua.gr

**Keywords:** cowpea, faba bean, common bean, BNF, organic, rhizobia, PGPR, green manure

## Abstract

An organic greenhouse crop of tomato was established in February following cultivation of cowpea (CP) or common bean (CB) for green pod production, or faba bean (FB) for green manuring. The vegetative residues of CP and CB were incorporated to the soil together with farmyard manure (FYM), prior to establishing the tomato crop. The FB plants were incorporated to the soil at anthesis together with either FYM or composted olive-mill waste (CO). Green manuring with FB resulted in higher soil mineral N levels during the subsequent tomato crop and higher tomato fruit yield when combined with FYM, compared to compost. The level of soil mineral N was the main restrictive factor for yield in organic greenhouse tomato. FB for green manuring as preceding crop to tomato increased significantly the level of soil mineral N and tomato yield compared to CB or CP aiming to produce green pods. The lowest tomato yield was obtained when the preceding crop was CB cultivated for green pod production. The soil mineral N was significantly higher when FYM was applied as base dressing compared with CO, despite the higher total N concentration in CO, pointing to slower mineralization rates of CO during tomato cultivation.

## 1. Introduction

The global market of organic products has increased more than three times during the recent 20 years due to commensurate increases in consumer demand for these products [1]. However, according to most researchers and practical experience, there is a yield gap of more than 20% between organic and conventional farming [2,3,4]. This difference in yield performance probably represents the most important limitation to the further expansion of organic farming [5].

Nitrogen (N) availability is of paramount importance for successful cultivation in any form of agriculture [6,7]. In organic farming, the use of industrially produced fertilizers of inorganic N is prohibited [3]. In addition, in many countries and throughout the European Union, the use of inorganic N in organic agriculture is not allowed, even if it is of mineral origin [8].

Providing N is a constant challenge for organic farming as it is the most important limiting factor for sufficient yields [9,10,11]. Overall, adequate N nutrition is difficult to achieve in organic farming and N deficiency often occurs [12]. Increasing the supply of N has a great potential to increase the yield in organic farming [13], since N inputs in organic farming are, in most cases, below the optimal level [5]. The main N sources in organic farming are the incorporation of green manure or crop residues and the application of animal manure or/and compost [6]. Nevertheless, the only true imports of N in a cultivated field are those originating from manure and compost transferred from other holdings, as well as atmospheric N_2_ fixed biologically by soil microorganisms [14].

Legumes, i.e., plant species belonging to the Fabaceae family, form symbiotic relationships with N_2_-fixing bacteria, generally termed rhizobia, and, therefore, legume-based green manure can provide significant amounts of N to the succeeding crops [15,16,17]. Several studies have shown that the use of legumes as green manure can effectively enhance the yield of the following tomato crop [17,18,19]. Different legume species are colonized by different rhizobial species. Therefore, when the appropriate rhizobial species for a particular legume crop are not present in the soil accommodating this crop, inoculation is necessary for sufficient biological N_2_ fixation that can effectively increases the total amount of produced biomass [19,20].

Crop rotations are the cornerstone of organic farming systems, as they contribute crucially to both maintenance of soil fertility [21], and control of pests, diseases and weeds [22]. Therefore, many scientists have proposed the reintroduction of legumes into the crop rotation systems, as they act as N fertilization sources for the next crop. Ponisio et al. [2] found that the application of crop rotation schemes based on legumes in organic farming reduces the aforementioned yield gap to 8%. However, the contribution of grain legumes to the soil N budget is limited, because roughly half of the biologically fixed N is removed by grain harvest [14], so that sometimes the final surplus may not exceed a level of 25 kg N ha^−1^ [22].

In this framework, the present study was carried out to test whether the growth, yield, and N nutrition of organic greenhouse tomato can be substantially improved when the preceding crop is a legume cultivated for harvesting green pods or as green manure. To attain this goal, tomato was organically cultivated in a greenhouse during spring-summer following autumn-winter cultivation of three alternative legume species. Two of the tested legume species (cowpea and common bean) were destined to produce green pods for the market, while faba bean was incorporated to the soil prior to anthesis as green manure.

## 2. Results

### 2.1. Legumes Biomass, Yield, N Accumulation and BNF

Common bean rendered significantly higher pod yield and plant residues compared to cowpea (Table 1). However, the aboveground fresh and dry biomass of cowpea or common bean were significantly lower than the corresponding fresh and dry faba bean biomass incorporated into the soil as green manure. This large difference was not due to the harvested pods in the common bean and cowpea crops, since their total biomass, including the pods, was significantly lower than that of faba bean. The pod harvest index of common bean was significantly higher than that of cowpea on fresh weight basis but similar between the two legumes when calculated on dry weight basis.

The N concentrations in the cowpea pods were higher than in those of common bean, whereas they were similar in the vegetative residues (Table 2). In both legumes, the N concentrations were higher in the pods than in the vegetative residues. The total N content, which is the product of concentration and total dry biomass, was substantially higher in both pods and vegetative residues of common bean compared to those of cowpea. However, the total N content in the faba bean biomass incorporated to the soil was about three times higher than in the total biomass of common bean and about seven times higher than in that of cowpea. The Ndfa in the cowpea biomass (39.6%) was significantly higher than in that of common bean (18.2%), but markedly lower than in the faba bean biomass (57%). Finally, the total amount of biologically fixed nitrogen in cowpea and common bean treatments was 1.51 and 1.65 g m^−2^, respectively, whereas it was appreciably higher in the faba bean treatment (15 g m^−2^). The N harvest index did not differ significantly between cowpea and common bean (44.8% and 43.4%, respectively).

### 2.2. Soil Measurements

The fertilization treatments applied to organic greenhouse tomato in the current study had no significant impact on the soil C, total N, P (Olsen) and exchangeable K concentrations throughout the experimental period (Table 3). After termination of all legume crops, the soil C, N, P, and K concentrations were slightly higher than those measured before commencement of their cultivation, but the differences were significant only for K and P. The subsequent cultivation of organic tomato did not reduce the soil C, total-N and P concentrations, but decreased significantly the soil K concentrations.

**Figure 1:**
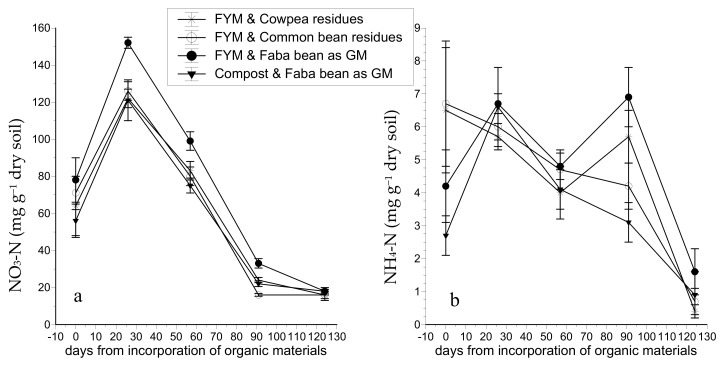
Impact of different organic fertilization treatments (**a**) on soil NO_3_-N concentrations; (**b**) on soil NH_4_-N concentrations during cultivation of organic tomato. FYM: farmyard manure; Compost: composted olive-mill waste; GM: green manure.

In the tomato crop, the soil NO_3_-N concentration increased considerably in all treatments 26 days after incorporation of the organic matter to the soil (DAIOM) but decreased gradually thereafter (Figure 1a). The incorporation of faba bean residues to the soil as green manure combined with farmyard manure (FYM) resulted in significantly higher soil NO_3_-N concentrations compared to the other three treatments at 26, 57, and 91 DAIOM. However, just before incorporation of the organic matter in the soil and at crop termination, the soil NO_3_-N was similar in all treatments. On the day of crop termination (at 124 DAIOM), the NO_3_-N concentration was below 19 mg kg^−1^ in all treatments.

The soil NH_4_-N concentration fluctuated strongly during the cropping period in the tomato crop, without significant differences between treatments (Figure 1b). Overall, the NH_4_-N concentration level was consistently low at all sampling dates and for all treatments ranging from 2 to 7 mg kg^−1^, while on the last sampling date, the NH_4_-N concentration dropped below 1.6 mg kg^−1^ in all treatments.

### 2.3. Tomato Yield Components

When faba bean applied as green manure was the preceding legume crop, the application of FYM as organic fertilizer increased the fruit yield in tomato, compared to application of composted olive-mill waste (Table 4). However, the fruit yield decreased significantly when the fertilization with FYM was combined with incorporation of cowpea or common bean residues to the soil originating from crops used for fresh pod production, compared to green manure with faba bean. Furthermore, the incorporation of common bean residues to the soil prior to planting further reduced the tomato yield compared to incorporation of cowpea residues. The reduction of tomato yield was exclusively due to commensurate decreases in the fruit number per plant, while the mean fruit weight was similar in all treatments.

### 2.4. Tomato Tissue Analysis

The highest leaf N concentration was measured in tomato plants fertilized with green manure of faba bean in combination with FYM (FB + FYM). When the green manuring with faba bean was combined with application of composted olive-mill waste, the leaf N concentration in tomato was significantly lower than that obtained from FB + FYM but similar to that obtained from application of FYM together with vegetative residues of cowpea cultivated for fresh pod production. Finally, incorporating vegetative residues of common bean to the soil reduced significantly the leaf N concentration in the subsequent tomato crop compared to incorporation of cowpea residues (Table 5). On the other hand, the leaf P and K concentrations were not influenced by any fertilization treatment in the organic tomato crop.

## 3. Discussion

### 3.1. Legumes Aboveground Biomass, Nitrogen Fixation and N Balance

In order to quantify the biologically fixed N, three values have to be measured, particularly total plant dry matter produced per m^2^, tissue N concentration (N %) and percentage of plant N derived from atmospheric N_2_. Of these three parameters, the dry matter production exhibits the larger variation between different crop species and cropping systems, and is, therefore the main factor that differentiates BNF in different legume crops [23]. In the present study, faba bean produced more than twice as much dry matter as common bean and more than five times that of cowpea. The major factor contributing to this large difference in dry biomass between faba bean and the other two legume crops was plant density, which was almost threefold in the faba bean plots destined for green manuring (11.7 plants m^−2^) compared to that applied in the crops of common bean and cowpea (4.27 plants m^−2^). In addition, the climatic conditions during the last two months of the legume crops were much more favorable for faba bean than for common bean and cowpea, as the greenhouse was not sufficiently heated (Table 5). Finally, the faba bean biomass was incorporated into the soil at the most appropriate stage, just before anthesis, while the residues of common bean and cowpea were incorporated after harvesting of all pods. The growth stage of legumes has also a substantial impact on the N accumulation in shoot [14,24]. Thus, at the time of incorporation into the soil, the N concentration in the faba bean tissues was significantly higher compared to those measured in common bean and cowpea residues, which were at a late reproductive stage. In addition to this, an appreciable amount of the total N contained in the plant biomass was removed through pod in the plots of cowpea and common bean, thereby further reducing the amount of N provided to the soil through incorporation of plant biomass. Thus, the amount of N provided to the soil through incorporation of plant biomass in the faba bean plots was ten times more than the corresponding amounts in the cowpea plots and five times more than in the common bean plots. The significant difference in total N provided to the soil by incorporation of common bean residues at crop termination compared to that provided by cowpea residues was solely due to commensurate differences in biomass production.

The %Ndfa ranged low in all treatments compared to values reported for the same legume species by Gatsios et al. [19], who worked in the same greenhouse soil, and by other investigators, who worked in different locations [25,26]. This is attributed to the high concentration of soil inorganic N, especially NO_3_, which ranged from 73 to 95 mg kg^−1^ when the legumes were sowed. High levels of plant available N (PAN) accumulated during soil solarization, which was applied for almost three months during the summer preceding the establishment of the legume crops. The high temperature and humidity conditions prevailing during soil solarization favored N mineralization, while the absence of plants during that time maintained the produced inorganic N available for the next crop. It is well documented [24,26,27] that high concentrations of inorganic N in the soil inhibit colonization of legume roots with rhizobia and concomitantly restrict symbiotic N_2_ fixation. Nevertheless, the %Ndfa of faba bean was significantly higher than that found in the other two legumes, presumably because of the high plant density of faba bean, as this crop was intended for application of green manure. Consequently, although the mineral N level in the soil was the same for all legumes at crop establishment, the amount of N relative to the total plant needs was less in faba bean and thus it was less restrictive for rhizobia colonization than in the other two legume species. An additional reason is that the BNF efficiency of faba bean is not particularly affected by the level of inorganic N in the soil, presumably because this plant has a relatively low efficiency to exploit the soil N [25,26,28]. Furthermore, the N_2_ fixation efficiency of common bean is generally lower than that of other legumes, according to several studies [21,23,26]. The present study confirmed the lower N_2_ fixation efficiency of common bean as the %Ndfa in this legume was appreciably lower than that found in cowpea and faba bean. The biologically fixed N per cultivated area unit that was provided to the next crop by incorporating faba bean to the soil as green manure amounted to 15 g m^−2^. This level is slightly lower than the 19 g m^−2^ found by Ntatsi et al. [29] in a crop cultivated for fresh pod production, but similar to that reported by Stagnari et al. [28]. Compared to the other two legumes, the amount of biologically fixed N_2_ accumulated by faba bean was ten times higher than that of cowpea and common bean, which was at a similar level. This is ascribed not only to the higher %Ndfa of faba bean but also to the markedly higher biomass produced by this legume.

Soil solarization increases the PAN levels in the soil due to the high temperatures that accelerate N mineralization, and thus its application after an N-demanding crop, such as tomato that depletes the PAN pool in the soil is highly beneficial. Thus, it can be applied in organically cultivated tomatoes in greenhouses. However, the release of PAN through soil solarization requires the presence of organic matter with a low C/N ratio in the soil. The present study showed that this could be achieved by the introduction of a legume crop in a yearly rotation scheme with tomato. The legume crop can be cultivated either for harvesting pods or as green manure, which is especially useful in infertile soils as has been reported by Thönnissen et al. [30]. In addition, legumes that maintain high %Ndfa even at high concentrations of PAN in the soil, such as faba bean, can be selected as shown in the current experiment and in previous studies [26,28]. Furthermore, at high PAN concentrations in the soil, the nodulation efficiency may be influenced also by the variety of a particular legume species which can interact with the rhizobial strains, as reported by Peoples et al. [26].

The dry matter harvest index of common bean and cowpea was rather low compared to that found in other studies [31,32] and this led to a low N harvest index (NHI). The final contribution of legumes to the N soil balance depends on the difference between the index of Ndfa index and the NHI. Therefore, in the case of common bean, in which the Ndfa index is appreciably lower than the NHI, the soil N balance was negative, while for cowpea it was neutral, or even slightly positive if also the root biomass is taken into account. The contribution of the below-ground legume biomass (root, exudates and nodules) to the N balance must be taken into account, as it is estimated to be about 30% of the above-ground biomass [25,26,33]. However, the N balance of the common bean remains negative even after taking into consideration the below-ground N contributed by symbiotic N fixation. Thus, the lower soil mineral N and fruit yield in tomato following common bean, compared to cowpea, is ascribed to the difference between N contributed by BNF and N removed through harvesting of pods. Indeed, this difference was strongly negative in common bean (1.65 vs. 3.68 g m^−2^), while in cowpea it was almost balanced (1.51 vs. 1.71 g m^−2^) and presumably slightly positive, if also the root biomass is taken into account. This means that the common bean crop removed part of the soil N reserves, while the cowpea crop retained the soil N reserves and presumably left some N from BNF to the following tomato crop. For this reason, the soil mineral N and the fruit yield were significantly lower in tomato following common bean, compared to tomato following cowpea, although common bean contributed more biomass to the soil than cowpea.

### 3.2. Soil Measurements

Ammonium concentrations were quite low in all samplings, and differences between all treatments were insignificant. This was expected because in nonacidic, well-aerated topsoils with high microbial activity, the nitrification rate of NH_4_ is high [34,35,36]. In contrast, NO_3_ concentrations were rather high before the incorporation of organic matter, indicating the partial exploitation of PAN by legumes. Indeed, as several studies have shown [26,32,37], the cultivation of legumes does not deplete the pool of inorganic N, as is the case with other plants and especially cereals. Under greenhouse conditions, nitrate leaching is unlikely because there is no rainfall and irrigation is completely controlled. The sharp increase in the soil NO_3_ concentration 26 DAIOM in all treatments reflects the high rates of mineralization that occur in the first weeks after incorporation of legume biomass to the soil [37,38,39]. However, the levels of NO_3_ were higher than the optimal range suggested for tomato cultivation in the literature [40,41]. At 57 DAΙOM, the NO_3_ concentration was within the optimal range, but at 91 DAIOM it dropped to lower levels than those suggested as sufficient for tomato cultivation [42,43,44]. In the latter critical period, the FYM + FB treatment maintained a significantly higher NO_3_ concentration than the other three treatments, while the FYM + CB treatment resulted in a significantly lower NO_3_ level compared to FYM + CP and CO + FB treatments. These results show that a higher amount of inorganic N was released through mineralization in the FYM + FB treatment compared to CO + FB, although the total N concentration in FYM was significantly lower than in CO. This is attributed to the much slower N mineralization rate in CO during the first year of application, which is estimated to 10% compared to 50% in FYM [45,46].

### 3.3. Tomato Growth and Yield

In agreement with the high level of soil PAN, tomato plants in the first stage of development showed symptoms of vigorous vegetative growth, which according to Papadopoulos [47] include curled thick leaves, thick stem with large diameter, and large clusters. Eight weeks after planting, tomato plants were balanced in terms of N nutrition, while in the third month of development they showed symptoms of N deficiency. This deficiency was mainly manifested by thin stems and light green leaves as described in relevant literature [40,47], while deficiency symptoms were mildest in the FYM + FB treatment.

The total N concentration measured in the leaves 2.5 months after planting was lower than the optimal values for tomato cultivation in all treatments, except for FYM + FB which was marginally lower [40,48]. These results were in line with the observed N deficiency symptoms that became visible at that cropping stage. The level of P and K concentrations in tomato leaves in all treatments was within the optimal range [40,48], as expected considering the concentrations of these elements in the soil.

The significant difference in the yield of tomato fruit between treatments is ascribed to PAN levels in the soil during the reproductive stage, when they became lower than those suggested for tomato, confirming that an adequate N supply is the most critical factor for organic cultivation [9,10,11]. Thus, the tomato yield in FYM + FB treatment was significantly higher than in the other three treatments, followed by FYM + CP, which is in line with commensurate differences in the soil NO_3_-N levels.

Several researchers [26,28,49] estimated that about 30% of the pre-crop effect of legumes on subsequent crop yield should be attributed to other factors beyond N supply, such as improvements in the physical, chemical, and biological properties of the soil. This effect could not be assessed in the present study. Thus, it is not possible to conclude if the yield differences were imposed only by differences in mineral N availability, or by other factors such as improvements in the physical, chemical, and biological properties of the soil. However, the lack of any impact of the different legume treatments on soil C and available K and P levels may indicate that physical, biological and chemical factors other than N mineralization did not have a measurable effect on the observed yield differences.

In organic farming, the most critical challenge is to synchronize the rate of N mineralization and plant N needs, as has been reported by many researchers [6,12,50]. In the present study, the data shown in Figure 1 and the visual appearance of the plants indicate that the N supply exceeded the N requirements of tomato during the first month of plant growth. This is ascribed to high N mineralization rates of legume biomass during the first 8 weeks after its incorporation into the soil, which are in agreement with previous reports [38]. However, after the first eight weeks, when the plants started to produce fruit, the soil mineral N levels decreased rapidly, suggesting that the rates of N mineralization were not sufficient to satisfy plant N uptake requirements. This lack in synchronization between N supply and N demand is crucial and needs to be mitigated in some way. Shortening the time interval between incorporation of the organic materials to the soil and planting of tomato might decrease both the peak in soil mineral N and the rate of the subsequent decrease, thereby maintaining sufficient soil N levels for longer time. In addition, the initial rate of plant biomass decomposition can be reduced by proper treatments such as adjusting the size of the shoot fragments or partially drying the biomass on the soil surface before its incorporation [12,51]. Moreover, legumes can be intercropped with other plants with a higher C/N ratio in order to reduce the initial rate of mineralization [14,37]. Finally, one should take into consideration that in drip-irrigated organic tomato crops in greenhouses, only a part of the organic biomass incorporated into the soil is utilized by the plants, as the drippers moisten constantly only an aliquot of the soil bulk. Thus, measures to moisten constantly additional parts of the soil at a later cropping stage might considerably enhance the soil N reserves that can be utilized by plants, thereby avoiding or minimizing yield restriction due to N deficiency. Nevertheless, this hypothesis has to be tested experimentally by modifying accordingly the irrigation system.

## 4. Materials and Methods

### 4.1. Plant Material, Growth Conditions, and Treatments 

An experiment with legumes as preceding crop and tomato as the main crop was conducted in a greenhouse NNE–SSW oriented, which was located in Preveza, northwestern Greece (38°59′29.2″ N; 20°45′36.1″ E, 5 m a.s.l.). The exact dates for each crop establishment, commencement of harvesting, and crop termination are provided in Table 6. Prior to the establishment of the experimental treatments, soil solarization was applied to control soil-borne pathogens. The soil solarization started on 13th June and lasted up to 5th September 2018. The experiment was carried out in a commercial arch type greenhouse with vertical sidewalls, covered by low-density polyethylene film. The geometrical characteristics of the greenhouse were as follows: eaves height = 2.80 m, ridge height = 3.5 m, span width = 7.5 m, length = 44 m, ground area = 330 m^2^. The greenhouse was ventilated via side vents (total opening area 150 m^2^), which were opened whenever the greenhouse air temperature exceeded 26 °C. The plot size was 3.75 × 5.00 m^2^ (i.e., 18.75 m^2^). The soil type was sandy loam with neutral pH (7.3 measured in water extract) and an organic matter content of 4.14%. The concentrations of plant available N (NO_3_-N and NH_4_-N) in the soil before the experiment are shown in Figure 1 (concentrations on day 0). The total N, P and K in the soil before the experiment are presented in Table 3.

During the experimental period, climatic data, particularly air temperature and relative humidity, were collected on an hourly basis. Monthly average temperature (mean, maximum, minimum) and relative humidity (%) values for all experiments are presented in Table 7.

In this experiment, four different treatments were established to test the impact of legumes cultivated as preceding crops on the succeeding organic tomato cultivation (Table 8). Specifically, in treatments 1 and 2, cowpea and common bean, respectively, were cultivated for harvesting fresh pods during autumn and winter of 2018. In treatments 3 and 4 faba bean was grown and incorporated into the soil before anthesis as green manure. Treatments 3 and 4 were identical at the stage of legume cultivation and differentiated afterwards by applying different sources of organic matter in each of them prior to establishment of the tomato crop.

In treatment 1, the seeds of cowpea were inoculated with *Bradyrhizobium* sp. VULI11 [52] and putative plant growth promoting rhizobacteria (PGPR). In treatment 2, the seeds of common bean were inoculated with *Rhizobium* sp. PVKA6 and PGPR, while in treatments 3 and 4 the seeds of faba bean were inoculated with *Rhizobium* sp. VFLE1 [53] and PGPR. In all treatments, the microorganisms applied as putative PGPR, which had been isolated from cowpea nodules were *Enterobacter* sp. strain C1.2, *Enterobacter* sp. strain C1.5, *Enterobacter* sp. strain C3.1, and *Lelliottia* sp. strain D2.4. Strain designations “C” and “D” represent the geographical regions of field-collected cowpea root nodules in Greece that are Epirus and Crete, respectively, and followed by a lab code number. Legume seeds were soaked for 2 min in gum arabic solution (20%) as adhesive to deliver 10^9^ cfu/mL of the microbial cell suspensions. For combined inoculation, the liquid cultures were mixed in equal proportions. The inoculated seeds were spread in the shade, air-dried for 12 h and sown in well-prepared soil.

The crop residues of cowpea and common bean in treatments 1 and 2, respectively, and the entire biomass of faba bean in treatments 3 and 4 were incorporated into the soil at the end of January 2019. In treatments 1, 2 and 3, farm-yard manure (FYM) originating from free-range cattle farming was applied on 1st February 2019 at a rate of 50 t ha^−1^. The FYM contained 0.34% N, 0.15% P, and 0.48% K. This amount of FYM was equivalent to a N supply of 170 kg ha^−1^, which is in compliance with the European Union Regulation 889/2008. In treatment 4, olive-mill waste compost was applied, containing 1.26% N, 0.08% P and 1.03% K, at a rate of 30 t ha^−1^, on the same date with FYM, which provided 378 kg N ha^−1^. Four replicates were applied in each treatment.

The tomato crop was established 20 days after incorporation of the legume biomass and FYM or compost to the soil. The commercial tomato hybrid ‘Nissos F1’ (Hazera seeds Ltd., Berurim M.P Shikmim, Israel) grafted onto the commercial rootstock ‘Maxifort F1’ (*Solanum lycopersicum* × *Solanum habrochaites*) was transplanted on 20th February 2019. All plants were pruned to a single stem and the plant density was 2.13 plants/m^2^. The tomato and legume plants were drip-irrigated. During the cropping period, no additional fertilizers were provided to the plants in all treatments.

### 4.2. Growth, Mineral Analysis, and Nitrogen Fixation by Legumes

Immediately after termination of the autumn-winter legume crops, their plant biomass was incorporated into the soil. The aboveground biomass incorporated into the soil included only the vegetative plant residues remaining after harvesting of the pods in the cowpea and common bean plots. However, in the faba bean plots, the entire plant biomass was incorporated to the soil as green manure. Before incorporation into the soil, the aboveground biomass was quantified by harvesting the shoots from an area of 1 m^2^ in each plot center and measuring their total fresh weight. The samples of the aboveground fresh biomass were oven-dried at 65 °C to a constant weight and weighed to determine their dry biomass. Subsequently, each dry biomass sample was homogenized and a subsample was collected, ground using a ball mill and sieved through a 40 mesh sieve to determine total-N and carbon (C) contents in plant tissue samples by high temperature combustion using an elemental analyzer (Unicube, Elementar Analysensysteme GmbH, Hanau, Germany).

The dry biomass data from the cowpea and common bean crops were further used to determine the pod harvest index (PHI) as a percentage of harvested pod biomass to the total biomass of both pods and vegetative residues. Similarly, the N harvest index (NHI) was calculated as the percentage of N accumulated in the pods relative to the total N accumulated in pods and residues.

The N derived from the atmosphere in the aboveground biomass of legumes was determined by applying a method based on the natural abundance of ^15^N in plant tissues relative to the air [19,54,55]. To apply this method, the stable N isotopic composition of legume tissue samples was determined using an Isoprime 100 continuous flow isotope ratio mass spectrometer coupled to a Vario Isotope Select elemental analyzer (Elementar Analysensysteme GmbH, Hanau, Germany). The δ-values were calibrated relative to air by means of a three-point calibration using standard reference materials IAEA-N1, IAEA-600, and IAEA-N2. Measurement uncertainty was monitored by repeated measurements of internal laboratory standards and standard reference materials. Precision was determined to be ±0.19‰ based on repeated measurements of calibration standards and internal laboratory standards. Accuracy was determined to be ±0.19‰ on the basis of the difference between the observed and known δ values of check standards and their standard deviations. The total analytical uncertainty was estimated to be ±0.27‰ for δ^15^N. The δ^15^N values were estimated as parts per thousand (‰) deviations relative to the nominated international standard of atmospheric N_2_ (0.3663%), using the following equation [56]:

(1) $$\delta^{15}N\left(‰\right)=\left(\frac{{\mathrm{atom}\%}^{15}\mathrm{Nsample}-0.3663}{0.3663}\right)\times 1000\;$$

Subsequently, the percentage of N derived from the atmosphere (%Ndfa) was estimated by substituting the δ^15^N (‰) of the N_2_-fixing legume and a non-N_2_-fixing reference plant grown in the same soil, as calculated using Equation (1), into the following equation suggested by Unkovich et al. [54]:

(2) $$\%\mathrm{Ndfa}=\left(\frac{\delta^{15}N\;\mathrm{of}\;\mathrm{reference}\;\mathrm{plant}-\delta^{15}N\;\mathrm{of}\;\mathrm{legume}}{\delta^{15}N\;\mathrm{of}\;\mathrm{reference}\;\mathrm{plant}-B}\right)\times 100$$

where “B” is the δ^15^N in shoots of cowpea, common bean or faba bean plants grown on an inert medium and starved of N throughout their life, thereby being fully dependent on N_2_ fixation. The B values used in the current study were −1.61 for cowpea, –2.16 for common bean and −0.50 for faba bean, as suggested by Unkovich et al. [54]. The reference plant used in this study to determine the corresponding δ^15^N values was the grass weed *Digitaria sanguinalis* (L.).

To determine the total amounts of biologically-fixed N_2_ by cowpea, common bean and faba bean per cultivated area unit (BNF, kg ha^−1^), the following equation was used [57]:

(3) $$\mathrm{BNF}=\frac{\mathrm{DB}\times \mathrm{Nt}\times \%\mathrm{Ndfa}}{100}$$

where DB is the total dry biomass of the shoot, Nt is the total N concentration (% *w*/*w*) in the aboveground dry biomass, and %Ndfa are the values obtained from (2).

### 4.3. Tomato Tissue Sampling and Mineral Analysis

The N, P and K nutrition of the tomato crop was estimated by collecting samples of the youngest fully expanded leaves from all plots of the experiment 2.5 months after planting. The leaves were washed with distilled water, chopped, and oven-dried at 65 °C until they reached constant weight, powdered using a ball mill, and passed through a 40 mesh sieve. Subsequently, 0.5 g of powdered material was dry ashed in a muffle furnace at 550 °C for 5 h, and the ash was dissolved in 1 N HCl. Phosphorus (P) was measured photometrically as phosphomolybdate blue complex at 880 nm using a spectrophotometer (U-2000, Hitachi, Tokyo, Japan). Potassium (K) was determined using a flame photometer (Sherwood Model 410, Cambridge, UK). Organic C and total N in plant tissue samples were determined as described above for the legume crops.

### 4.4. Soil Analysis

Soil samples were collected from the central square of each plot (dimensions 2 × 2.5 m^2^). In each plot, 5 soil cores weighing about 400 g were collected from the root zone of 5 plants at a depth of 0–20 cm. Samples were prepared according to Miller et al. [58] and analyzed to determine the total N, NO_3_-N, NH_4_-N, and plant-available P and K concentrations. Total N in soil samples was determined by high temperature combustion using an elemental analyzer (Unicube, Elementar Analysensysteme GmbH, Hanau, Germany). To determine the concentration of mineral N (N-min, i.e., NO_3_-N + NH_4_-N) in the soil, each sample of sieved soil was extracted using a KCl solution, as described by Keeney and Nelson [59]. Subsequently, the NO_3_ and NH_4_ concentrations in the sample extracts were determined by applying the cadmium reduction to NO_2_ and the indophenol blue methods, respectively [59], using a Spectronic Helios spectrophotometer (Thermo Electron Corporation, Mercers Row, Cambridge CB5 8HY, UK). Plant-available P was determined using the Olsen method [60] and quantified by molybdate colorimetry [61]. Exchangeable soil K was determined using a flame photometer (Sherwood Model 420, Sherwood Scientific, Cambridge, UK) following extraction with an ammonium acetate solution.

### 4.5. Tomato Production and Yield Components

The impact of the experimental treatments on crop yield was assessed by harvesting all ripe tomatoes from 10 plants of the plot center twice per week and recording their number and total weight.

### 4.6. Statistical Analysis

The experiment was set as randomized block designs with 4 treatments and 4 replicates per treatment. The data were statistically analyzed by applying ANOVA using the STATISTICA software package, version 12.0 for Windows. The Duncan’s multiple range test was applied to separate means when the ANOVA was significant at *p* < 0.05. Data are presented in graphs and tables as means ± SE of four replicates.

## 5. Conclusions

In organic greenhouse tomato, a pre-crop of legumes can provide additional N to the crop, as the high N demands cannot be fully covered by application of animal manure due to restrictions in the maximum allowed amounts imposed by EU legislation. Common bean aiming to produce green pods does not provide substantial amounts of N, as its efficiency to fix N_2_ symbiotically is low. Hence, the amount of N removed by harvesting green pods is higher than that fixed symbiotically. Cowpea is more efficient in symbiotic N fixation, while its potential for green pod production is lower, and thus it may leave some of the symbiotically fixed N to the next crop. However, the best option proved to be the cultivation of faba bean as green manure, which can successfully supplement or even substitute farm-yard manure in some cultivation plans. In all treatments, the %Ndfa in the current study was lower than the potential levels, due to the relatively high mineral N concentrations in the soil before sowing legumes, originating from intensive mineralization during summer, when soil solarization was applied.

The compost of olive-mill waste did not adequately replace farm-yard manure as it provided lower amounts of soil mineral N during tomato cultivation that led to lower fruit yield.

The lack of synchronization in N supply through legume biomass mineralization and N demand by tomato was confirmed in the present study. During the vegetative and the initial reproductive stage of tomato there was an excess in soil mineral N. However, the mineral N levels dropped to lower levels than the optimal range during the latter cropping stage in all treatments, although this reduction was milder in the faba bean plots. The yield performance was commensurate with the levels of mineral N at the late cropping stage of tomato. This result indicates that the mineral N level is the main restrictive factor for yield performance in organic greenhouse tomato, which has a longer harvesting period than open-field crops. Practices increasing the exploitation of the organic matter incorporated to the soil as N fertilizer source in greenhouse organic tomato might prevent N deficiencies at the late cropping stages and enhance yield performance.

## Figures and Tables

**Table 1:** Aboveground fresh and dry biomass of harvested pods (BHP), biomass incorporated to the soil (BIS) after crop termination, total produced biomass (BTP), and harvest index (HI) in autumn-winter crops of common bean and cowpea used for fresh pod production, and faba bean applied as green manure.

Treatment	BHP (g m^−2^)	BIS (g m^−2^)	BTP (g m^−2^)	HI (%)
	Fresh biomass			
Cowpea	450 b	885 c	1335 c	33.7 b
Common bean	1660 a	2048 b	3708 b	44.8 a
Faba bean	-	7015 a	7015 a	-
Significance	***	***	***	*
	**Dry biomass**			
Cowpea	43 b	79 c	122 c	35.2
Common bean	108 a	203 b	311 b	34.7
Faba bean	-	681 a	681 a	-
Significance	***	***	***	ns

Means (n = 4) followed by different letters within each column indicate significant differences according to the Duncan’s multiple range test (*p* < 0.05); *, *** significant at *p* < 0.05 and *p* < 0.001, respectively; ns = not significant.

**Table 2:** Total-N concentrations in plant tissues, total-N provided to the next crop by incorporation of the legume biomass to the soil, N harvest index (NHI), percentage of N derived from the atmosphere (Ndfa %) in the legume biomass incorporated to the soil, and total amount of biologically fixed N (BNF) per unit area cultivated with a legume.

Treatment	Tissue N (%)		Total N Content (g m^−2^)			NHI %	Ndfa %	BNF (g m^−2^)
	Residues	Pods	Residues	Pods	Total			
Cowpea	2.67 b	3.97 a	2.11 b	1.71 b	3.82 c	44.8	39.6 b	1.51 b
Common bean	2.64 b	3.41 b	5.36 a	3.68 a	9.04 b	43.4	18.2 c	1.65 b
Faba bean	3.93 a	-	-	-	26.42 a	-	57.0 a	15.0 a
Significance	***	*	***	***	***	ns	***	***

Means (n = 4) followed by different letters within each column indicate significant differences according to the Duncan’s multiple range test (*p* < 0.05); *, *** significant at *p* < 0.05 and *p* < 0.001, respectively; ns = not significant.

**Table 3:** Impact of different organic fertilization treatments on concentrations of organic C, total-N, available P (Olsen), and exchangeable K in the soil. FYM: Farmyard manure; CO: composted olive-mill waste.

Treatment	C %	N %	P mg kg^−1^	K mg kg^−1^
	**Prior to starting the experiment**			
**FYM + Cowpea residues**	2.48	0.265	86.6	811
**FYM + Common bean residues**	2.33	0.273	91.3	832
**FYM + Faba bean green manure**	2.41	0.270	86.3	811
**CO + Faba bean green manure**	2.36	0.260	92.1	784
**Significance of differences**	ns	ns	ns	ns
	**After tomato planting**			
**FYM + Cowpea residues**	2.70	0.275	113	1038
**FYM + Common bean residues**	2.63	0.281	116	1086
**FYM + Faba bean green manure**	2.57	0.283	122	1196
**CO + Faba bean green manure**	2.59	0.278	111	1141
**Significance of differences**	ns	ns	ns	ns
	**At tomato crop termination**			
**FYM + Cowpea residues**	2.64	0.264	109	825
**FYM + Common bean residues**	2.55	0.271	110	853
**FYM + Faba bean green manure**	2.62	0.271	116	976
**CO + Faba bean green manure**	2.59	0.269	105	880
**Significance of differences**	ns	ns	ns	ns

Means (n = 4) followed by different letters within each column indicate significant differences according to the Duncan’s multiple range test (*p* < 0.05); ns = not significant.

**Table 4:** Impact of different organic fertilization treatments on fruit yield and yield components in the organic greenhouse tomato crop. FYM: Farmyard manure; CO: composted olive-mill waste.

Treatment	kg m^−2^	Fruit Number per Plant	Mean Fruit Weight
**FYM + Cowpea residues**	14.5 b	28.3 b	241
**FYM + Common bean residues**	11.4 c	22.8 c	235
**FYM + Faba bean green manure**	15.8 a	30.4 a	244
**CO + Faba bean green manure**	13.9 b	27.1 b	240
**Significance of differences**	***	***	ns

Means (n = 4) followed by different letters within each column indicate significant differences according to the Duncan’s multiple range test (*p* < 0.05); *** significant at *p* < 0.001; ns = not significant.

**Table 5:** Impact of different organic fertilization treatments on leaf N, P, and K concentrations in the organic greenhouse tomato crop. FYM: Farmyard manure; CO: composted olive-mill waste.

Treatment	N mg g^−1^	P mg g^−1^	K mg g^−1^
**FYM + Cowpea residues**	27.4 b	3.21	71
**FYM + Common bean residues**	25.2 c	3.25	72
**FYM + Faba bean green manure**	29.8 a	3.27	78
**CO + Faba bean green manure**	27.6 b	3.04	79
**Significance of differences**	**	ns	ns

Means (n = 4) followed by different letters within each column indicate significant differences according to the Duncan’s multiple range test (*p* < 0.05); ** significant at *p* < 0.01; ns = not significant.

**Table 6:** Dates of crop establishment, commencement of harvesting, and crop termination for the legume and the tomato crop.

	Establishment	Start of Harvesting	Crop Termination
Cowpea	09/12/2018	11/22/2018	01/31/2019
Common bean	09/12/2018	11/02/2018	01/31/2019
Faba bean	09/25/2018	-	01/31/2019
Tomato	02/20/2019	05/21/2019	07/15/2019

**Table 7:** Monthly averages for mean, maximum, and minimum daily temperatures (Tmean, Tmax and Tmin, respectively) and relative humidity (RHmean, RHmax and RHmin, respectively) inside the greenhouse during the experimental period (2018–2019) in Preveza, Greece.

Month	T_mean_	T_max_	T_min_	RH_mean_	RH_max_	RH_min_
**June 2018**	25.5	33.1	20.1	62.3	84.6	33.9
**July 2018**	26.7	34.4	21.1	59.5	80.1	30.2
**August 2018**	28.8	38.3	22.9	61.2	82.9	32.0
**September 2018**	24.7	33.4	20.6	65.4	84.9	39.6
**October 2018**	22.4	31.6	18.0	65.2	83.6	37.5
**November 2018**	17.4	26.9	12.6	77.4	98.8	50.5
**December 2018**	12.9	24.2	8.1	84.9	100	54.8
**January 2019**	10.2	22.6	5.5	85.1	99.1	58.5
**February 2019**	15.9	27.9	8.1	65.5	86.0	27.9
**March 2019**	16.5	28.5	9.9	70.4	88.7	37.6
**April 2019**	18.4	27.5	12.6	79.2	99.5	44.9
**May 2019**	19.6	30.7	13.0	76.6	96.0	40.3
**June 2019**	25.1	34.5	17.9	74.1	96.7	41.4
**July 2019**	26.3	35.1	21.4	72.3	95.5	42.8

**Table 8:** Description of the treatments applied to test the impact of legumes as preceding crops to organic tomato cultivation.

No	Treatment Short Name	Treatment Description
**Legume Crop**		
1.	CP	Cowpea (*Vigna unguiculata* (L.) Walp.) inoculated with *Bradyrhizobium* sp. VULI11 and PGPR ^1^ and cultivated for fresh pod production
2.	CB	Common bean (*Phaseolus vulgaris* L.) inoculated with *Rhizobium* sp. PVKA6 and PGPR ^1^ and cultivated for fresh pod production
3.	FB	Faba bean (*Vicia faba* L.) inoculated with rhizobia (*Rhizobium* sp. VFLE1) and PGPR ^1^ and incorporated to the soil as green manure
4.	FB	Faba bean (*Vicia faba* L.) inoculated with rhizobia (*Rhizobium* sp. VFLE1) and PGPR ^1^ and incorporated to the soil as green manure
**Tomato Crop**		
1.	FYM + CP	Farmyard manure (FYM) & cowpea crop residues
2.	FYM + CB	FYM & common bean crop residues
3.	FYM + FB	FYM & faba bean green manure
4.	CO + FB	composted olive-mill waste & faba bean green manure

1: A mix of *Enterobacter* sp. C1.2, *Enterobacter* sp. C1.5, *Enterobacter* sp. C3.1, and *Lelliottia* sp. D2.4.

## Data Availability

Data is contained within the article.
